# Editorial: Disruptors on Male Reproduction - Emerging Risk Factors

**DOI:** 10.3389/fendo.2022.934098

**Published:** 2022-05-31

**Authors:** Yankai Xia, Honggang Li, Rossella Cannarella, Panagiotis Drakopoulos, Qing Chen

**Affiliations:** ^1^ State Key Laboratory of Reproductive Medicine, Center for Global Health, School of Public Health, Nanjing Medical University, Nanjing, China; ^2^ Institute of Reproductive Health, Tongji Medical College, Huazhong University of Science and Technology, Wuhan, China; ^3^ Department of Clinical and Experimental Medicine, University of Catania, Catania, Italy; ^4^ Centre for Reproductive Medicine, Universitair Ziekenhuis Brussel, Vrije Universiteit Brussel, Brussels, Belgium; ^5^ Department of Obstetrics and Gynaecology, University of Alexandria, Alexandria, Egypt; ^6^ In Vitro Fertilisation (IVF) Athens, Athens, Greece; ^7^ Key Lab of Medical Protection for Electromagnetic Radiation, Ministry of Education of China, Institute of Toxicology, College of Preventive Medicine, Army Medical University (Third Military Medical University), Chongqing, China

**Keywords:** reproduction, risk factors, lifestyle & behaviour, environmental factors, genetic susceptibility

Over the past decades, male reproductive health has been deteriorating, partially due to the exposure to environment and lifestyle harmful factors (1, 2). With emerging and widespread harmful substances in our daily life, it becomes urgent to identify and assess their risk on male reproductive health.

Certain genetic variants could increase the susceptibility of the reproductive system to the environmental damage (3). The field of genomics has provided an extraordinary level of knowledge, aided by large-scale, unbiased genome-wide association studies (GWAS). A similar level of analysis, however, is still lacking for the influences of environmental factors on the reproductive phenotype (4, 5).

Never before, in human history, has there been such a vast multiplicity of environmental risk factors, nor has there been such expression of concern regarding their effects on health, especially on reproductive health (6). Increasing scientific evidence has shown the adverse impacts of environmental risk factors on human reproduction (7). Maternal or paternal exposure to environmental chemicals (e.g., pesticides, heavy metals, phthalates, and polycyclic aromatic hydrocarbons) can lead to a myriad of health consequences, which can manifest across individuals’ lifespan and potentially be transmitted to future generations (8).

Although the risk factors of traditional environmental pollutants have been intensively investigated, their contributions could only explain a limited proportion of the reproductive damages. On the other hand, in modern society, emerging factors including novel physical factors (e.g., C-irradiation, cryopreservation, Wi-Fi), environmental chemical exposures (e.g., TiO_2_ nanoparticles, PM_2.5_, per-fluoroalkyl and polyfluoroalkyl substances, pesticides), biological contamination (e.g., COVID-19), behavioral and lifestyle factors (e.g., pornography use, circadian desynchrony, assisted reproductive technology) and diseases status [e.g., type 2 diabetes mellitus, obstructive sleep apnea (OSA)] have not been studied in detail. To be noted, these factors have been even less studied with regard to male reproductive damages compared to female disorders. It is urgent to understand these novel factors in terms of populational distribution/burden, impacts on male reproductive health (endocrinal disruption, sperm damage, subfecundity and infertility) as well as the underlying mechanisms.

This Research Topic aims to provide insights into the contribution of novel environmental, lifestyle and psychological factors to male reproductive damages and the mechanisms. In Volume I we have accepted 18 articles and reviews, which provide interesting and exciting insights to this growing field with coverage of various potential risk factors.

Radiation is a ubiquitous environmental exposure in modern society. In the current Research Topic, Guo et al.  investigated that the abscopal effects of C-irradiation on testis with regard to both structure and function and ultimately decreased sperm quality in mice. Chen et al.  studied the motility, acrosomal integrity, and mitochondrial membrane potential (MMP), as well as proteomic change, of cynomolgus macaque sperm after cryopreservation. They hypothesized that AFP III may reduce the release of cytochrome C and thereby reduce sperm apoptosis by modulating the production of ROS in mitochondria. This may represent a novel molecular mechanism for cryoprotection. In addition, Jaffar et al.  concluded that the long-term Wi-Fi exposure from pre-pubertal to adult age could reduce spermatogonia proliferation in the testis.

Another well celebrated example of risk factors was exposure to environmental chemicals. Although *in vitro*, Mancuso et al. highlighted the adverse effects even of subtoxic dose of TiO_2_ nanoparticles on porcine prepubertal Sertoli cells (SCs) functionality and viability and, more importantly, set the basis for further *in vivo* studies, especially in chronic exposure at subtoxic dose which is closer to the human exposure to this nano agent. Calvert et al. reviewed the literature on the biological effects of per-fluoroalkyl and polyfluoroalkyl substances (PFAS) exposure, with a specific focus on male reproduction, owing to its utility as a sentinel marker of general health. Shi et al. demonstrated that PM_2.5_ exposure could induce spermatocyte damage and energy metabolism disorder. The activation of the aryl hydrocarbon receptor might be involved in the mechanism of male reproductive toxicity. Gao et al. suggested that a manipulation on the expression of signaling proteins regulating spermatogenesis could possibly be used to manage the toxicant-induced male reproductive dysfunction.

Environmental factors mediate changes in expression patterns can be explained by a complex network of modifications to the DNA, histone proteins and degree of DNA packaging as well as changes in DNA structure such as mitochondrial DNA copy number and chromatin integrity. Throughout our lives, epigenetic processes shape our development and enable us to adapt to a constantly changing environment. Ma et al. integrated glufosinate-ammonium (GLA) induced alterations in sperm epigenome and embryo transcriptome, and further explored their concordance, thus providing a new strategy for gamete-to-embryo toxicity assessment. Intriguingly, this study also noted that paternal GLA exposure induced aberrant transcription in both paternal and maternal alleles of preimplantation embryos, which deserves further investigation. Wang et al. concluded that bivalent chromatin structure resulted in large differences in the methylation of autism genes between manually selected spermatozoa (MSS) and Zona pellucida (ZP)-bound spermatozoa (ZPBS). Intracytoplasmic sperm injection (ICSI) using MSS, which increased the risk of methylation changes compared with ZPBS, may lead to a higher risk of autism in offspring. Liu et al. contributed to the understanding of epigenetic regulation of tet methylcytosine dioxygenase 1-Sp1 transcription factor (TET1-SP1) during spermatogonia self-renewal and proliferation. Shi et al. focused on the associations between sperm mitochondrial DNA copy number (mtDNA-CN), DNA fragmentation index (DFI), and reactive oxygen species (ROS) and embryo development as well as pregnancy outcomes in assisted reproductive technology (ART).

COVID-19 is a serious challenge to the global health systems. It has been found that the hazardous effects of COVID-19 go far beyond respiratory system, and a body of studies explored the impact of COVID-19 on male reproduction from different aspects. Guo et al. reviewed the relationship of COVID-19 and male reproduction and provided a panoramic view to understand the effect of the virus on male reproduction and a new perspective of further research for reproductive clinicians and scientists.

Behavioral and lifestyle factors may also have a substantial contribution to the damage of male reproductive health. As suggested by Cui et al., pornography use was common among male college students in China. Early contact, frequent use, and high frequency of masturbation during pornography use could lead to not only addiction trends but aberrant reproductive hormone levels and semen quality as well. Up to date, this article has received over 6,700 views and exceeded 83% of all the Frontiers articles in only 7 months. Fusco et al. aimed to provide data about pre-clinical and clinical evidence exploring the impact of circadian desynchrony on spermatogenesis. Zatecka et al. emphasized the importance of improving metabolic health not only in women of reproductive age, but also in potential fathers, in order to reduce the negative impacts of diabetes on subsequent generations. Feng et al. characterized erectile dysfunction (ED) in OSA patients.

Updated information about increasingly emerging environmental perpetrators, including physical, chemical, biological and behavioral/lifestyle risk factors can improve our understanding of male reproductive health. With the rapid evolvement of omics technologies in genomics, epigenomics, transcriptomics, proteomics and metabolomics, it is reasonable to expect that in the near future there will be plenty of studies to renew our knowledge on emerging risk factors and the attendant adverse effects on male reproductive health, as well as the underlying mechanisms. Hence, to keep the constant concern and to inspire new studies to this filed, the Volume II focusing on the same topic has started and we anticipate to receive high-quality submissions all over the world (https://www.frontiersin.org/research-topics/35111/).

## Author Contributions

All authors listed have made a substantial, direct and intellectual contribution to the work, and approved it for publication.

## Funding

The work was supported by the National Key Project of Research and Development Program (Grant number: 2018YFC1004200, 2018YFC1004202) and the Ability Promotion Programme of Army Medical University (Grant number: 2021XZL01).

## Conflict of Interest

The authors declare that the research was conducted in the absence of any commercial or financial relationships that could be construed as a potential conflict of interest.

## Publisher’s Note

All claims expressed in this article are solely those of the authors and do not necessarily represent those of their affiliated organizations, or those of the publisher, the editors and the reviewers. Any product that may be evaluated in this article, or claim that may be made by its manufacturer, is not guaranteed or endorsed by the publisher.
